# The Prospects of Electromagnetic Stimulation in Cartilage and Bone Tissue Engineering

**DOI:** 10.3390/cells15040325

**Published:** 2026-02-10

**Authors:** Ivan V. Zhivodernikov, Stanislav Y. Ershov, Karina D. Goncharova, Tatiana V. Kirichenko, Yuliya V. Markina, Alexander M. Markin

**Affiliations:** 1 Petrovsky National Research Centre of Surgery, 119435 Moscow, Russia; 2 Petrovsky Medical University, 119435 Moscow, Russia; 3 Medical Institute, Peoples’ Friendship University of Russia Named After Patrice Lumumba (RUDN University), 117198 Moscow, Russia

**Keywords:** electromagnetic field, MSCs, osteogenic differentiation, chondrogenic differentiation

## Abstract

**Highlights:**

**What are the main findings?**

- Electromagnetic fields stimulate cell differentiation, in particular osteogenic and chondrogenic MSC differentiation.
- Extracellular vesicles can be considered as mediators of electromagnetic stimulation, suggesting that electrically stimulated cells may produce exosomes with enhanced regenerative properties.

**What are the implications of the main findings?**

- Electromagnetic stimulation is a complementary or alternative tool in the classical tissue engineering triad that includes cells, scaffolds, and biochemical stimuli for increasing chondro- and osteogenesis.

**Abstract:**

The achievements of regenerative medicine are based on methods of controlling stem cell division and differentiation. Electromagnetic fields stimulate cell differentiation by means of affecting calcium channels and cellular signaling. However, only a small part of the mechanisms underlying electromagnetic field effect on cells has been studied. The prospect of their use in tissue engineering as an addition or alternative to biochemical effects becomes clear in the course of numerous experiments. Electromagnetic stimulation enhances the effect of biochemical differentiation inducers and can cause the secretion of exosomes of special properties, which may serve as a therapeutic tool. For example, it has been shown that EMFs at 15 Hz and 2 mT increased the expression of chondrogenic differentiation markers SOX9 and COL2 in human bone-marrow MSCs by up to 3-fold (based on Parate et al.). Optimizing EMF parameters (e.g., 15–50 Hz, 1–2 mT) for specific cells and pathologies remains a key challenge of the studies in the field of tissue engineering. This review describes the electromagnetic field effect on the chondrogenic and osteogenic differentiation of MSCs of various origins, which is important for the musculoskeletal tissue recovery, as well as on inflammatory diseases in model animals.

## 1. Introduction

Electric fields are involved in diverse physiological processes including nerve conduction, tissues regeneration, and direct somatic embryogenesis [1]. Various electric field stimulation systems have been designed to study the effects of electric stimulation on cells functions in vitro. The methods of electric stimulation of cultured cells can be divided into three types: direct coupling, capacitive coupling, and inductive coupling using an electromagnetic field (EMF) [2]. The EMF is generated by a conductive coil placed around the cell culture system, and the stimulation is transmitted by the pulse to mimic the natural potential transfer in the organism. This is a significant advantage of this stimulation type, despite the significant technical difficulties compared to other types of electrical stimulation.

The electromagnetic exposure influences the morphology, orientation, migration, and phenotype of multiple types of cells. The EMF also stimulates cellular differentiation across a wide range of parameters [3]. The degree of a cell’s relationship to a particular type of tissue determines the tissue functionality and its microenvironment development. The studies of electromagnetic influence on cell processes have shown some therapeutic prospects for tissue and cellular engineering [4].

According to current studies, the therapeutic effect of electromagnetic fields depends on different parameters such as intensity, frequency, duration, and amplitude, as well as the shape of the electromagnetic wave or its changes over time. The researchers employ complex combinations of parameters: from very-low-frequency EMFs, which may cause osteogenic or chondrogenic differentiation of stem cells [5,6], to radiofrequency EMFs, which suppress tumor growth [7,8]. It is necessary to control the intensity of an EMF during the treatment since the high intensity of the EMF stimulates the cells to produce more energy that can lead to cell growth and metabolism deceleration [9]. That is, the intensity of the electromagnetic field appears to be an important parameter for regulating EMF effects. Electromagnetic therapy is considered as a universal therapeutic approach that has been tested in a large number of in vivo and in vitro studies, as well as clinical trials [10,11,12,13].

**Figure 1:**
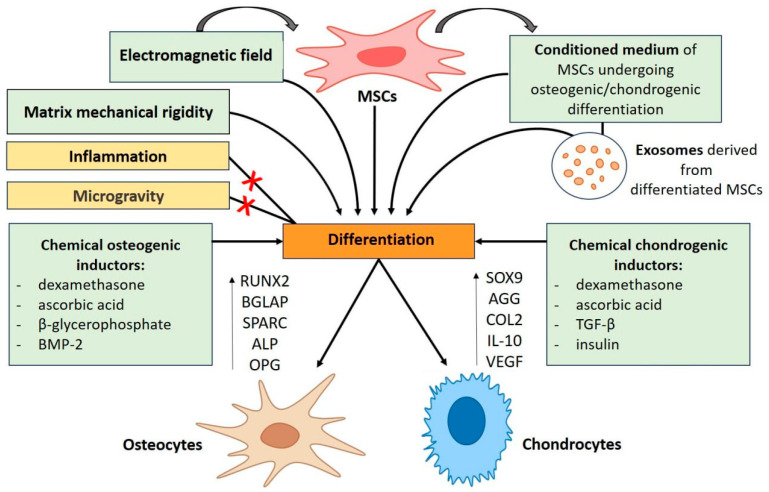
Factors inducing differentiation of MSCs. AGG—aggrecan; ALP—alkaline phosphatase; BMP-2—bone morphogenetic protein 2; BGLAP—bone gamma-carboxyglutamic acid-containing protein (osteocalcin); COL2—type 2 collagen; IL-10—interleukin-10; MSCs—mesenchymal stem cells; OPG—osteoprotegerin; RUNX2—runt-related transcription factor 2; SOX9—SRY-box transcription factor 9; SPARC—secreted protein acidic and cysteine rich (osteonectin); VEGF—vascular endothelial growth factor.

Electromagnetic therapy is applied for treating a wide range of diseases associated with impaired regeneration (e.g., bone fracture non-union and osteoporosis) and repairing cartilage and tendons in osteoarthritis recovery. Furthermore, it was shown in several studies that the EMF helps to recover bone and cartilage defects in various experimental animal models, suggesting the need to develop the mentioned approach. Extracellular vesicles are increasingly studied in the field of regenerative medicine and tissue engineering since they are safer and more controllable therapeutic agents than cell transplantation [14]. At the same time, exosomes are considered as emerging mediators of electromagnetic stimulation effects, suggesting that electrically stimulated cells may produce extracellular vesicles with enhanced regenerative and anti-inflammatory properties [15]. Figure 1 demonstrates the factors inducing osteogenic and chondrogenic differentiation.

The current review summarizes the results of the studies exploring the effects of electromagnetic fields on cellular chondrogenic and osteogenic differentiation, intracellular signaling pathways, and cartilage and bone regenerative outcomes to discuss the potential of biophysical stimulation within the classical tissue engineering triad that includes cells, scaffolds, and biochemical stimuli, positioning electromagnetic fields as complementary or alternative tools to enhance chondro- and osteogenesis. The literature search was based on the analysis of publications containing the keywords “electromagnetic field, exosomes, mesenchymal stem cells (MSCs), chondrogenic and osteogenic differentiation” in the PubMed and Scopus databases up to December 2025.

## 2. EMF and Chondrogenic Differentiation

Cartilage tissue is slowly regenerated in a natural way due to the low cell content in the intercellular substance and nutrients infiltration via diffusion. The cartilage repair rate is much lower than that of other tissues, and the approaches to increasing the cartilage regeneration are currently studied [16]. Cartilage consists of the collagen protein, which has piezoelectric properties; this fact makes cartilage tissues and chondrogenic cell cultures very attractive for research with the use of EMFs [17]. Transforming growth factors such as TGF-β, which relate to the morphogenetic protein (BMP) family, stimulate the morphogenesis of cartilage tissue. They are expressed during skeletal development, chondrogenesis, and fracture recovery. Under artificial conditions, this process is modeled in vitro using biochemical agents. Several laboratories have demonstrated that exposure to electromagnetic fields leads to activation of members of the TGF-β/BMP family in MSCs or IPSC cultures, as well as in human fractures [18,19]. TGF-β activates SRY-box transcription 9 (SOX9), a transcription factor that leads to increased synthesis of collagen and aggrecan proteins which are characteristic of the extracellular matrix of cartilage tissue.

In the study of Feiyuan Qiu et al., the differentiation of MSCs exposed to EMF or biochemical chondrogenic inducers was compared. The EMF groups were exposed to 50 Hz, 1 mT EMFs for 30 min every day for 10, 15, and 20 days separately. Another experimental group was cultured in a chondrogenic medium, while the control group was cultured in a nutrient medium lacking chondrogenic inducers. The type 2 collagen (COL2) and aggrecan (AGG) genes and the corresponding proteins expression levels were significantly higher when exposed to EMF or chemical stimulation in comparison with the control group, despite the fact that the chemical inducers exceeded the effect of EMFs. Nevertheless, it was shown that low-frequency EMFs stimulate rat BM-MSCs to differentiate into chondrogenic cells [20]. Parate et al. revealed the greatest effectiveness of the exposure to bone-marrow MSCs under a single 10 min electromagnetic field set to 2 mT and 15 Hz. A lower (1 MT) or higher (>3 MT) EMF amplitude, as well as shorter (5 min) or a longer (>20 min) exposure, led to a decrease in the effect. Interestingly, repeated exposure reduced the chondrogenic effect, and this decrease was eliminated by blocking EGTA or TRP channels TRPC1 and TRPV4. The study highlights the importance of calcium homeostasis within early chondrogenesis and shows the possibilities and limitations of electromagnetic fields for future methods of cartilage tissue regeneration [21].

Since exposure to an electromagnetic field has a stimulating effect on chondrogenic and osteogenic differentiation, it is used as a countermeasure in experiments having negative effects on osteo- and chondrogenesis. An example of such an influence is micro- or zerogravity. Many studies show that the presence of an organism or cell culture in zerogravity makes it difficult for cells to differentiate in chondrogenic and osteogenic direction. On the scale of the body, it leads to accelerated bone resorption and a decrease in its strength. In experiments to study the cellular and molecular mechanisms of this phenomenon, cell cultures are subjected to simulated microgravity in terrestrial conditions in order to study countermeasures eliminating the effects of microgravity on the whole organism. Such an experimental model using special bioreactors was employed by Wagner et al. in the studies of chondrogenesis in a three-dimensional culture of human MSCs. An SMG bioreactor which had an external motor drive was combined with Helmholtz magnetic coils to create an electromagnetic field (5 MT; 15 Hz).

Pellets derived from human mesenchymal stem cells were cultured under control conditions or exposed to microgravity, electromagnetic fields, or a combined influence for 3 weeks. The simulated microgravity conditions reduced the expression of the COL2 alpha 1 chain (COL2A1) and AGG genes. Changes were not detected in the experimental group which was exposed only to the electromagnetic field. In the group exposed to mixed factors, the expression of the same genes increased compared to in the group exposed to microgravity. This fact indicates the restoration of chondrogenic potential [22]. Kavand et al. observed gene expression within MSCs differentiation in a three-dimensional alginate structure when exposed to an electromagnetic field set at frequencies of 25 and 50 Hz. Six groups of cell−alginate constructs were tested and treated for 21 days. According to real-time PCR data, the treatment with TGF-β had a greater effect on the growth of COL2 and SOX9 gene expression compared to the treatment only with EMFs. It has been shown that COL2 expression is more sensitive to EMFs [23]. In the study by Yu et al., an increase in the content of Col2, Sox9, and Agg proteins was observed in the 3D culture of MSCs from mice carrying magnetic nanoparticles [24]. Mayer-Vanger et al. found that exposure of cultures to low-frequency electromagnetic fields (15 Hz, 5 MT) for 45 min every 8 h increased COL2 expression and glycosaminoglycan content, but it did not affect the expression of AGG or SOX9. During electromagnetic stimulation, the expression of the COL10 gene decreased [25].

In addition to studies of MSC differentiation, the effect of EMFs on chondrocytes in vitro has been described. When stimulating human chondrocytes, which were obtained as a result of knee arthroplasty, a dose-dependent cell viability was found. The highest viability and production of extracellular matrix components were recorded after exposure to an EMF set at a frequency of 0.1 Hz and a voltage of 1.95 µT for a duration of 60 min per day over 3 days. Exposure to an EMF with a voltage of 0.65 µT and a frequency of 1 Hz led to a less pronounced effect. The effect of electromagnetic fields having a strength of 1.3 µT and a frequency of 10 Hz had no significant effect on various analytical parameters [26]. The study of in vitro chondroprogenitor cells cultivation compared the effects of the EMF and TGF-β on chondrogenic differentiation [27]. Parate et al. reported modulation of the chondrogenic MSC secretome upon 10 min exposure to an EMF with an intensity of 0.5–4 mT and a duration of 6 ms, repeated at a frequency of 15 Hz [28]. M De Mattei et al. studied the dose-dependent effect of several EMF parameters on proteoglycan synthesis in articular cartilage explants, depending on the field intensity, frequency, and duration of exposure. It has been shown that field intensity is a significant factor in the range from 0.5 to 2.0 MT; frequency is a significant factor in the range from 2 to 110 Hz, while the difference between the four doses is minimal; duration of exposure leads to a significant increase in the amount of proteoglycan in the range from 4 to 24 h [29].

While in vitro cellular systems make it possible to study isolated cellular reactions, in vivo animal studies make it possible to preserve cells and intercellular connections within a three-dimensional tissue. In the context of differentiation, in vivo models provide an opportunity to observe enchondral ossification, which occurs in embryogenesis, bone growth zones, and recovery from fractures. One of these models is the model of enchondral ossification of the demineralized bone matrix (DBM-EO), a fragment of which is filled with MSCs after implantation. Studies using this model have demonstrated that TGF-β, a key growth factor necessary for chondrogenic differentiation, is synthesized in cells being exposed to an EMF [30]. The property of the EMF to stimulate the synthesis of TGF-β, which, in turn, is an inducer of chondrogenic differentiation, reveals one of the mechanisms of the chondrogenic effect of the EMF.

The production of TGF-β during chondrogenesis was described with the use of the DBM-EO model and an EMF (5 ms, 15 Hz) [31]. A significant increase in the concentration of TGF-β protein was observed in osteogenic cells stimulated by the EMF, compared to the control group on day 2 and during early calcification (days 8–10). The physiological peak of TGF-β on day 6 was observed in bones stimulated by the EMF, and it increased within exposure. Immunohistochemical tests showed that the synthesis of TGF-β was carried out primarily by chondrocytes, rather than by MSCs. It is important to note that, with the cessation of chondrogenesis and the onset of early calcification, the content of glycosaminoglycans, as well as the concentration of TGF-β, decreased. It demonstrates that TGF-β concentrations correspond to stimulation and cessation of chondrogenesis [32]. The mechanisms of the EMF effect on chondrogenic differentiation are associated with the redistribution of calcium ions. In the process of chondrogenic differentiation, calcium ions play a key role in the activation of TGF-β [33,34]. Electromagnetic fields change the membrane potential, activate calcium channels and, as a result, cause fluctuations in calcium concentration and consequently affect TGF [35,36]. This contributes to the differentiation of stem cells and chondroprogenitors in the direction of chondrogenesis [37,38]. The main participants are the transient receptor potential cation channel subfamily C member 1 (TRPC1) and transient receptor potential vanilloid type 4 (TRPV4) channels. In addition, an increase in interleukin-10 (IL-10) and vascular endothelial growth factor (VEGF) during exposure to an EMF is also important.

Table 1 presents the results of the studies aimed to investigate the effects of EMFs on MSC chondrogenic and osteogenic differentiation.

## 3. EMF and Osteogenic Differentiation

The electromagnetic field can promote the differentiation of MSCs into osteogenic progenitor cells and osteoblast differentiation, which, along with chondrogenic differentiation, is important for joint treatment. Runt-related transcription factor 2 (Runx2), core-binding factor a1 (Cbfa1), and osterix (Sp7) are the well-known predominant factors of transcription and osteogenic differentiation of MSCs, while Sox9 and modulation of the Wnt/β-catenin signaling pathway control chondrogenesis in cell cultures. The increased expression levels of osteoblast marker genes Runx2 and osteocalcin and other markers of osteogenic differentiation are observed in MSCs exposed to EMFs, as well as the expression of Sox9, Col2a1, and Agg is typical for chondrogenesis [49,50].

However, the main quantitative indicators of differentiation efficiency vary, depending on the parameters of electrical stimulation and cultivation conditions [51,52]. Kang et al. showed the dependence of the activity of alkaline phosphatase (ALP) and the expression of osteomarker genes, depending on the frequency of the current at an equal induction value of 1 Mt. At the frequency of 45 Hz, the electromagnetic field strongly stimulated the expression of RUNX2 in MSCs from human adipose tissue in comparison with the control and other experimental groups. At a frequency of 30 Hz, the electromagnetic field also increased the expression of RUNX2 compared to the control group. However, exposure to an electromagnetic field with a frequency of 7.5 Hz led to results similar to the control group lacking exposure, and in the case of expression of the osteocalcin gene, it led to a decrease in comparison in the control group. The frequencies of 60 and 75 Hz were also less effective than 30 and 45 Hz [44].

Enert et al. exposed adipose tissue MSCs, which were co-cultured with osteoblasts in different ratios, to an EMF with an average intensity of 140 µT at two frequencies of 16 and 26 Hz. The chosen EMF modes were also tested on osteoclasts derived from peripheral blood mononuclears. After 7 and 14 days of the experiment, the best result of ALP activity and matrix mineralization was shown at the ratio of osteoblasts and ad-MSCs of 3:1, compared to monocultures, and the both chosen frequencies modes were equally effective. Osteoclast activity (Trap5B expression) was better affected by a current of 26 Hz. Thus, a lower frequency current (16 Hz) is effective for bone formation, and a higher frequency current (26 Hz) is effective for remodeling stimulation. The study is of great interest for modeling the formation and resorption of bone tissue, since the co-cultivation model simulates cells at varying degrees of differentiation in the bone remodeling unit (BMU), and the assessment of osteoclast activity under EMF exposure allows making assumptions about resorption. The study is limited by the ratios of cell populations which are known at the start of the experiment, but not at the control points of days 7 and 14, whereas these ratios may change [46]. Lim et al. studied the effect of the ultra-low-frequency electromagnetic fields on the proliferation and differentiation of mesenchymal stem cells derived from human alveolar bone (hABMSCs). The study examined the effect of low-frequency EMFs on cell proliferation, alkaline phosphatase activity, and extracellular matrix mineralization, as well as the expression of vinculin, vimentin, and calmodulin in HABMSCs during osteogenic differentiation.

On day 5, EMF stimulation of cells increased proliferation by 15% compared to the control group. Furthermore, ultra-low-frequency EMFs significantly increased ALP expression in the early stages of osteogenesis and significantly improved mineralization later. In comparison with the control group, the EMFs positively affected the expression of vinculin, vimentin, and calmodulin, as well as genes associated with osteogenic differentiation [47]. In Yang et al.’s experiment, low-frequency EMFs (50 Hz, 20 MT) were used. It did not significantly affect hMSCs for 23 days, but the MTT test showed inhibition of proliferation and metabolism. It should be noted that differentiation is an energy-consuming process, and even if there were not any significant changes in ALP activity and mineralization, the conditions resulting in reducing these indicators were registered [9].

The pERK and Wnt/β-catenin pathways play an important role in the osteogenic differentiation of MSCs or osteoblasts, and calcium ions have a significant effect on both pathways during the differentiation [53,54]. EMFs affect the permeability of potential-dependent channels and promote calcium ions influx [55,56], thereby stimulating osteogenesis of stem cells. However, the mechanism of calcium ion effect on the Wnt/β-catenin signaling pathway may be more complex and involve interaction between calcium ions and the cellular cilia system [57]. It has been shown that the ability of EMFs to activate the Wnt10b/β-catenin signaling pathway and stimulate osteogenic cell differentiation depends on the primary cilia in osteoblasts. When the activity of primary cilia was suppressed by miRNA, the Wnt10b/β-catenin signaling pathway was no longer activated, and the ability of the electromagnetic field to stimulate osteogenic differentiation decreased significantly [58,59].

Nitric oxide (NO) acts as a messenger molecule, activates the cGMP and protein kinase G and pathways, and participates in increasing ALP expression in cells and their differentiation towards osteogenesis [60]. The concentration of nitric oxide (NO) is sensitive to EMFs; moreover, calcium channel inhibitors inhibit the production of NO [61]. The concentrations of NO and calcium ions inside the cell have a direct correlation; therefore, the variations in NO concentration may represent another mechanism by which EMFs affect cell differentiation [62]. EMF-induced NO production, in addition to differentiation, reduces the secretion of pro-inflammatory cytokines [63,64].

Chemical inducers of MSC differentiation into cells with osteogenic profile are calcium glycerophosphate, dexamethasone, ascorbic acid, and BMP (most commonly BMP-2). The introduction of these inducers in various combinations and concentrations leads to the formation of an extracellular matrix saturated with collagen 1, mineralization, and expression profile of genes which are peculiar to osteoblasts (mainly the transcription factor RUNX2 and the genes ALP, bone gamma-carboxyglutamic acid-containing protein (BGLAP)–osteocalcin, osteoprotegerin (OPG), secreted protein acidic and cysteine rich in cysteine (SPARC), also known as osteonectin, etc.) [65,66].

When studying osteogenic processes with the use of cellular models, researchers often resort to mechanical stimulation of osteodifferentiation in order to increase its effectiveness. The significant role of mechanical action on adherent cells in osteogenic differentiation is known. It can be the shear stress generated by the nutrient medium current in the perfusion system, when the calcification process of the vessel walls is simulated, or from direct pressure applied to the adherent layer using cellular substrates. It is also common to grow cells on synthetic cellular scaffolds, which are subsequently subjected to stretching or compression, or act mechanically due to their structure [67,68,69].

It is known that cell adhesion molecules are associated with signaling pathways responsible for the activation of the RUNX2 transcription factor, and modulation of mechanical stress in matrices and scaffolds activates the signaling of AMPK, Wnt, FAK, ERK1/2, Rho-ROCK, as well as the Smad-dependent signaling pathway TGF-β/BMP signaling pathways responsible for osteogenic differentiation [70,71,72]. When studying the osteogenic effects of electromagnetic fields, in addition to chemical inductors, researchers often introduce a matrix with special mechanical properties into the cellular model as an additional factor.

The synergistic effect of electromagnetic fields combined with other stimulating elements has been thoroughly studied. The effects of an EMF (0.2 Mt, 15 Hz) and biochemical stimulation on osteogenic differentiation of MSCs were studied by Jazayeri et al. in vitro and in vivo. After 10 days of EMF stimulation for 6 h per day, it was found that the combination of chemical inducers and the EMF improved osteogenesis. Furthermore, it has been shown in animal models that the use of differentiated osteoblasts placed on collagen scaffolds promotes the formation of new bone tissues [40].

As a model for the study of osteoporosis, Ye et al. printed porous titanium scaffolds which had a pore size that simulated bone tissue, while an EMF was used as an exogenous stimulus for osteogenesis induction (50 Hz; 1 MT; 2 h per day). The EMF increased the expression of the Alp, Runx2, and Bmp-2 genes on the surface of titanium scaffolds, which improved the osseointegration of tissue-engineered structures in a rabbit osteoporosis model [41]. Aldebs et al. found that exposure to a low-frequency pulsed EMF (15 Hz, 1 MT) for 8 h per day over 21 days improves the osteogenic potential of human MSCs (hASCs) cultivated on a three-dimensional hydrogel framework containing paramagnetic iron oxide nanoparticles [45]. Mirzai et al. found that the presence of a conductive polymer can enhance the positive effect of magnetic fields on the osteogenic development of dental pulp MSCs. According to the data, polyaniline and an EMF enhance the ability of MSCs for osteogenic differentiation [73].

The results show that the use of an EMF having this workload mode causes early phases of bone formation. Wang et al. studied the effect of electromagnetic fields (15 Hz/1 MT) on osteogenic differentiation of rabbit bone-marrow MSCs on a hydroxyapatite/collagen I scaffold. The study found that low-frequency electromagnetic fields improve osteogenic cell differentiation, and xenograft obtained using an EMF was more effective in bone tissue recovery in a rabbit femoral condyle defect model [48]. Habib et al. stimulated MSCs using TEM (15 Hz) for 4 h daily over 10 days. During the process, alkaline phosphatase activity and expression of BMP-2, BGLAP, and SPP1 genes increased faster when combined with a tricalcium phosphate bone substitute doped with iron ions.

The obtained results demonstrate the combined effect of an EMF and mentioned polymer on osteogenesis, and it suggests that this combination can be used to restore bone tissue [42]. In the study, Shaoyu Wu et al. studied the effect of an EMF on osteoblastogenesis of bone-marrow MSCs of rats labeled with paramagnetic iron oxide nanoparticles (SPIONs). BMMSC rats labeled with SPIONs were exposed to a low-frequency pulsed EMF with a frequency of 50 Hz at a voltage of 1.1 Mt. Exposure to the EMF led to increased proliferation and osteogenic differentiation of SPION-labeled BMMSCs compared with the control group, which was revealed by means of von Kosse staining and the use of alkaline phosphatase of cells (ALP). Both results suggest that the combination of an EMF and SPIONs has a synergistic effect on stimulation of directed migration and osteogenic differentiation [74].

Thus, the modal mode of an EMF in cellular models for stimulating osteogenic differentiation is about 50 Hz and 1 Mt, although frequencies from 10 to 70 Hz and induction from 0.2 to 20 mT are also effective. Exposure time varies from 30 s to 8 h per day. Apparently, there are minimum thresholds that trigger the necessary signal cascades, and the relationship of the effectiveness of the values to the magnitude quickly reaches a plateau. Nanoparticles which have magnetic properties are also used to stimulate osteogenic differentiation along with nanocomposite scaffolds; however, their properties are mainly revealed experimentally. Many mechanisms by which nanoparticles stimulate differentiation remain unknown, although it is known that some of them are involved in signaling pathways or have antioxidant properties [20,75,76,77,78]. It makes them promising components for therapeutic bone healing solutions, in which the effectiveness of EMFs has already been shown in animal models.

Despite the fact that osteogenic differentiation exposed to EMFs significantly increases in cellular models, the results of therapy employing pulsed electromagnetic fields are more restrained. Martinez-Rondanelli et al. reported a slight acceleration in the recovery of femoral diaphysis fractures of patients treated with EMP compared with the placebo group. In 12 weeks after the start of treatment, fracture fusion occurred in 75% of cases, compared with 58% in the placebo group. After 18 weeks, these values were 94% and 80%, respectively, and after 24 weeks, these values were 94% vs. 87%, respectively. It should be understood that the most significant and significant differences relate to earlier follow-up periods, since over time the EMF effect on bone integrity is smoothed out by natural bone tissue regeneration. Hannemann et al. did not find any significant effect of EMFs on the fusion of navicular bone fractures during the observation period. Mohajerani et al. did not find any significant increase in the bone density of the mandible in 4 weeks either. However, the percentage of bone density changes of two groups showed that bone density increased significantly 4 weeks after surgery in the treated group compared with the control group [79]. The gap between beneficial experimental results and clinical outcomes requires further research of the effectiveness of using EMFs for regenerative medicine. While in vitro studies show promising results, clinical trials demonstrate only modest improvements, for example, 75% versus 58% bone union at 12 weeks [80]. Nevertheless, the results of in vitro experiments indicate that EMFs are a possible option for use in tissue engineering bone recovery.

## 4. Cellular Exosomes After Electrostimulation: A New Way of Differentiation?

Exosomes derived from MSCs or other cells have been recently considered promising for therapy, along with autologous or allogeneic MSC transplantation. Exosomes are 40–150 nm extracellular vesicles which are produced by most cell types and participate in intercellular communication, capsulating proteins and microRNAs. Exosomes constituents reflect the condition of the cells which they are secreted from; nonetheless, it has been confirmed the introduced exosomes have an impact on recipient cell cultures and experimental animals. The exosomes originating from various sources (MSCs, endothelial cells, other chondrocytes, and platelet-rich plasma) are also known to affect chondrocytes [81,82]. Their certain origin may promote chondrocytes proliferation, migration, and differentiation and reduce joints inflammation in vivo. The recent studies of cellular differentiation showed that the conditioned environment of differentiated cells induces differentiation itself. The cell exosomes, as well as soluble secreted proteins, loaded with microRNAs and growth factors, also impart this property to the nutrient medium [83,84]. Experiments have shown that the addition of exosomes derived from mature chondrocytes can stimulate cell differentiation or improve cartilage health condition.

Weekley administration of mouse chondrocyte exosomes over 12 weeks significantly improved cartilage tissue repair [85]. Promoting cartilage tissue regeneration, it is important to manipulate with the matrix component, since the major part of the cartilage consists of the extracellular matrix (ECM), which retains water. Exosomes derived from MSCs stimulate extracellular matrix deposition, enhancing the synthesis of collagen II and proteoglycans. At the same time, they reduce the expression of enzymes which lyse the extracellular matrix: ADAMTS-5, matrix metalloproteinases (MMPs), and collagenase [86]. Exosomes from embryonic stem cells also have a stimulating effect on the collagen II synthesis [87]. Given that mentioned exosomes properties impact chondrogenesis, some researchers have modified them to increase their effect by enhancing expression or loading target molecules. San et al. found that BMSC exosomes, which overexpress miR-320c, stimulate chondrocyte proliferation more effectively, inhibit MMP13 production and enhance Sox9 expression [88]. Another study of miR-150-5p via the Wnt5A signaling pathway showed similar results [89]. The injection of exosomes containing miR-9-5p into the joints of rats suffering osteoarthritis reduced the content of pro-inflammatory factors such as IL-1, IL-6, and TNF-α.

Thus, it is assumed that the exosomal miR-9-5p microRNA suppresses inflammation and oxidative stress, thereby improving the health condition of osteoarthritis in rats. Attempts to find out which microRNAs may be responsible for the chondrogenic effect of exosomes are also being made. Ma et al. showed, in mice with osteoarthritis, the anti-inflammatory effect of exosomes derived from bone-marrow MSCs is associated with miR-205-5p [90]. The mechanisms governing the sorting of biochemical agents into exosomes have not been studied; however, it has been repeatedly confirmed that the effect of exosomes on cells or tissues directly depends on the source of the exosomes. It is also known that stimuli impacting exosome donor cells are an important factor for pro-inflammatory, anti-inflammatory, and differentiation effects. Therefore, there are many experimental approaches based on obtaining exosomes which have certain properties after the exposure on their source. In the case of cellular differentiation, exosomes derived from committed cells stimulate differentiation of recipient cells in the same direction. To obtain chondrogenic and osteogenic exosomes, standard biochemical inducers, growth factors, cartogenin, and mechanical stimulation were tested as differentiation inducers.

Exosomes derived from committed cells and obtained by these types of induction stimulated the differentiation of recipient cells [91,92]. Most experiments involving cell culture stimulation by an electric current or EMF set with certain parameters have shown stimulating effects on chondrogenic and osteogenic differentiation. Thus, exosomes derived from differentiated MSCs by electrical stimulation can be expected to differentiate other MSCs. Such issues have emerged relatively recently and mainly consider chondrogenic differentiation [83,93]. Yang et al. describe the production of exosomes by adipose-derived MSCs of healthy rats stimulated by a pulsed electromagnetic field with an amplitude of 1 mT and a frequency of 15, 45, and 75 Hz. The obtained exosomes inhibited staurosporin-induced apoptosis of chondrocytes, and the most effective were exosomes derived from MSCs stimulated at a frequency of 75 Hz. A year later, the same research team published the results of the study with a similar experimental design, but the exosome recipients were rats with simulated osteoarthritis. The exosomes of MSCs exposed to an EMF suppressed inflammation and degeneration of cartilage, as evidenced by a higher content of COL2A1, SOX9, and ACAN, and a lower expression of MMP13 and caspase-1 compared to the control group [83,93]. Since exosomes are in most cases positioned as a promising therapeutic tool, their positive effects in a therapeutic context (in particular, regenerative and anti-inflammatory processes) are more often reported. However, there are experiments showing exosome-mediated pro-inflammatory activation. For example, Yang et al. found that exosomes secreted by IL-1b-simulated vascular endothelial cells inhibit autophagy, reduce the ability of chondrocytes to withstand oxidative stress and may contribute to the development of arthritis. The effects of exosomes derived from EMF-stimulated MSCs on chondrogenic and osteogenic differentiation are presented in more detail in Table 2.

It can occur indirectly through the signaling pathways of IL-1b, IGF, and NF-kB, and microRNAs may be one of the links in such reactions [94]. Exosomes, which were isolated from the synovial fluid of patients with osteoarthritis, stimulated macrophages to release pro-inflammatory cytokines [88]. On the one hand, it illustrates exosomes as a diagnostic tool for the study of inflammatory biomarkers in blood or synovial fluid and, on the other hand, as a promising method for the treatment of osteoarthritis and cartilage degeneration [95]. The use of electrical stimulation or an EMF can expand the functionality and accessibility of such a method, as it can complement the effect of biochemical induction or, in some cases, serve as an alternative.

Exosomes are extracellular vesicles carrying miRNAs and proteins that can replicate cell transplant benefits. What goes inside these exosomes depends on the treatment the donor cells: EMF exposure to adipose MSCs prevents chondrocyte death and reduces osteoarthritis inflammation by increasing COL2A1/SOX9 while decreasing MMP13. Other triggers (biochemical or mechanical) also produce useful exosomes, but EMFs offer an accessible enhancement method though there is potential for unwanted pro-inflammatory effects.

## 5. Conclusions

The combination of cells that stimulate growth signals and polymer scaffolds possessing desired properties is often referred to as the “triad of tissue engineering”. To create a tissue engineering structure or increase the regeneration rate of damaged musculoskeletal tissue, a combination of additional biochemical signals and an extracellular matrix of specified mechanical properties is necessary. The electromagnetic field can be considered as an inductor that enhances the effect of biochemical stimuli and can be an additional tool for managing cells of tissue engineering structures during differentiation, along with growth factors and exosomes. Three-dimensional matrices composed of biocompatible polymer materials will provide a structural basis for maintaining cell adhesion and proliferation, which ultimately leads to tissue development [96].

The main limitation of the current studies aimed to access the effects of the EMT on cultured cells is the fact that technical aspects of the stimulation are often misreported, which is believed to hold the field back. Furthermore, the cell culture medium consists of water and dissolved salts, so a part of the energy is dissipated as heat interacting with these polar molecules. For the same reason, an EMF is refracted and shielded, limiting the depth of penetration into the culture medium. These properties are more common for high-frequency electromagnetic waves such as microwaves and radio waves. Pulsed EMFs at these frequencies are not used in electrical stimulation, but many experiments employ EMFs with inductions ranging from a few to several tens mT. Therefore, when comparing experimental data, the depth of the culture medium and the position of the cells relative to the EMF source should be considered if possible. However, most published studies do not provide such data. The comparison of experimental data in this field is also complicated by the large number of device types, many of which are designed by researchers themselves.

Articular cartilage is a particularly attractive target for tissue engineering strategies. Most approaches to its recovery and regeneration are based on cellular technologies and are aimed at creating a population of chondrogenic cells at the injury site. The cells used to develop these strategies are either MSCs from other anatomical locations capable of differentiating into chondrocytes or differentiated chondrocytes isolated from intact areas of the articular surface. Such cell sources do not seem to be a good choice, since taking a tissue biopsy from valuable healthy articular cartilage will eventually lead to new injuries, as the cartilage takes quite a long time to repair. Therefore, a lot of research efforts have been devoted to the mechanisms of MSC differentiation and ways to accelerate it [97].

Nowadays, research is underway to find and combine optimal modes of electrical stimulation of different cell types and other ways of their differentiation. The standardization and uniformity of the methodology is a difficult task, without which scalability of application in clinical practice is impossible, since many researchers show results with different EMF parameters. Therefore, before the time when such treatments become publicly available, it is necessary to ensure safety and effectiveness through follow-up studies and risk assessment management [98]. Future research should focus on randomized controlled trials using standardized 15–50 Hz/1–2 mT parameters and explore AI-based approaches for optimizing treatment protocols.

## Figures and Tables

**Table 1:** The effects of EMFs on MSC chondrogenic and osteogenic differentiation.

Object	EMF Mode	Exposure Time	Effect	References
Rat bone-marrow-derived MSCs	1 mT 50 Hz	30 min/day 10–20 days	Increased expression of Col2 and Agg	[39]
	Extremely-low-frequency pulsed EMF: 0.1, 0.2 * mT 15 Hz	40 min exposure + 27 min interval 6 h/day 2, 4, and 10 * days	Osteogenesis enhancement in explants: increased expression of Runx2 and osteocalcin	[40]
Human bone-marrow-derived MSCs	1, 2 *, 3, and 4 mT 15 Hz	5, 10 *, 20, 30 min, one time	Increased expression of *SOX9*, *COL-II*, and *AGG*	[21]
	5 mT 15 Hz	45 min exposure + 390 min interval 3 times/day 21 days	Restoration of *COL2A1*, *COLXA1*, and *AGG* gene expression lowered by simulated microgravity	[22]
	0.5, 1, 2, 3 *, 4 mT 15 Hz	10 * and 30 min barrages of 20 × 150 μs on−off pulses for 6 ms	*COL2A1*, *COLXA1*, and *AGG* gene expression restoration	[28]
	1 mT 50 Hz	2 h/day 14 and 21 * days	Enhanced alkaline phosphatase, *RUNX2*, osteocalcin, *BMP-2*	[41]
	EMF mode: square wave with a 3.85 kHz pulse frequency, a slew rate of 10 T/s 15 Hz	4 h/day 10 days	Enhanced alkaline phosphatase and increased expression of *BMP-2*, *BGLAP*, and *SPP1*	[42]
3D MSC−alginate construct	1.6 mT 25 * and 50 Hz	8 h/day 21 days	Increased levels of Col2, Sox9, and Agg proteins	[23]
Human MSCs derived from thighbone and tibia of a 12-week-old spontaneously aborted fetus	20 mT 50 Hz	23 days	The growth and metabolism inhibition	[9]
Human adipose-derived MSCs	100–250 µT 10–90 Hz (26 * Hz)	7 min one time	Akt и MAPK/ERK signal pathway activation	[43]
	1 mT 7.5, 30 *, 45 *, 60, 75 Hz	2, 4, 6 * h/day 10 days	Increased expression of *RUNX2* and *SPARC* genes	[44]
Human adipose-derived MSCs on a 3D construct	1 mT 15 Hz	8 h/day 7, 14, and 21 * days	EMFs induced early differentiation of hAD-MSCs in to an osteoblastic phenotype compared with cells without biophysical stimulation.	[45]
Co-culture of human adipose-derived MSCs and osteoblasts/osteoclasts	6–282 µT 16 and 26 Hz	7 min/day 5 times/week 7 and 14 * days	16 Hz: enhanced alkaline phosphatase activity and calcium content 26 Hz: osteoclast activation (increased secretion of TRAP5b)	[46]
Human alveolar bone-derived MSCs	600 µT 10, 50 *, and 100 Hz	72 h	Increased alkaline phosphatase expression during the early stages of osteogenesis and substantially enhanced mineralization	[47]
Rabbit bone-marrow MSCs on a hydroxyapatite/collagen I scaffold	1 mT 15 Hz	4 h	Bone regeneration within the defect and bone integration between the graft and host bone	[48]
Articular cartilage explants	0.5, 1, 1.5 *, and 2.0 mT 2, 37, 75, and110 Hz	1, 4, 9, and 24 * h (the difference between the modes was non-significant)	Increased proteoglycan synthesis	[29]

*, the most effective pulsed EMF mode.

**Table 2:** The effects of exosomes derived from EMF-stimulated MSCs on chondrogenic and osteogenic differentiation.

Exosome Source	EMF Settings	Main Results	Reference
Rat adipose MSCs	1 mT, 75 Hz	Reduced apoptosis, elevated COL2A1/SOX9	[83,93]
BMSCs with miR-320c	N/A (biochemical)	Better proliferation, lower MMP13	[88]
OA synovial fluid	None	Increased inflammatory markers	[88]
Mouse MLO-Y4	Mechanical mechanical tensile strain of 2500 με at 0.5 Hz for 1 h per day for 3 days	Enhanced MC3T3-E1 osteogenic differentiation	[92]
human umbilical cord mesenchymal stem cells (UC-MSCs)	(3D) scaffold using tricalcium phosphate nanoparticles (triCaPNPs)	Enhanced UC-MSC osteogenic differentiation	[91]
Chondrogenic BMSCs	None	Inhibition of inflammation in mice with arthritis	[90]
Human BMSCs	miR-320c overexpression	Enhanced Sox9 expression, MMP-13 inhibition	[88]
Mouse BMSCs	miR-150-5p overexpression	MMP-14 inhibition, reduction of inflammation in a mouse model of arthritis	[89]

## Data Availability

No new data were created or analyzed in this study.
